# Purpuric Lesion in a Newborn

**DOI:** 10.1177/00099228231196744

**Published:** 2023-08-30

**Authors:** Megan Schmalz, Ionela Iacobas, Priya Mahajan

**Affiliations:** 1 Department of Pediatrics, Baylor College of Medicine, Houston, TX, USA; 2 Cancer and Hematology Center, Texas Children’s Hospital, Houston, TX, USA

**Educational Objectives**

- Recognize kaposiform hemangioendothelioma (KHE) as a potential cause of a growing purpuric lesion in children.
- Understand the workup and management of KHE.
- Recognize Kasabach-Merritt phenomenon as a lethal coagulopathy associated with rare vascular tumors, such as KHEs.

## Case Report

A 4-week-old female, born at 38 weeks of gestational age via spontaneous vaginal delivery, presented to clinic with concern for a mass on the left leg. The lesion was first noted at birth and rapidly enlarged in size. Review of systems was negative for bleeding, fussiness, fever, decreased appetite, or changes in urine or stool output. The pregnancy was complicated by maternal hypertension and diabetes mellitus requiring insulin in the last month of pregnancy. Routine screening for infections during pregnancy was negative. Family history was unremarkable.

The infant was well appearing, with the following vitals: temperature of 99.4°F, respiratory rate of 42 breaths/min, heart rate of 142 beats/min, and oxygen saturation of 99% on room air. Her weight was 3861 grams (25th percentile), increased from a birth weight of 3520 grams.

**Figure 1.**
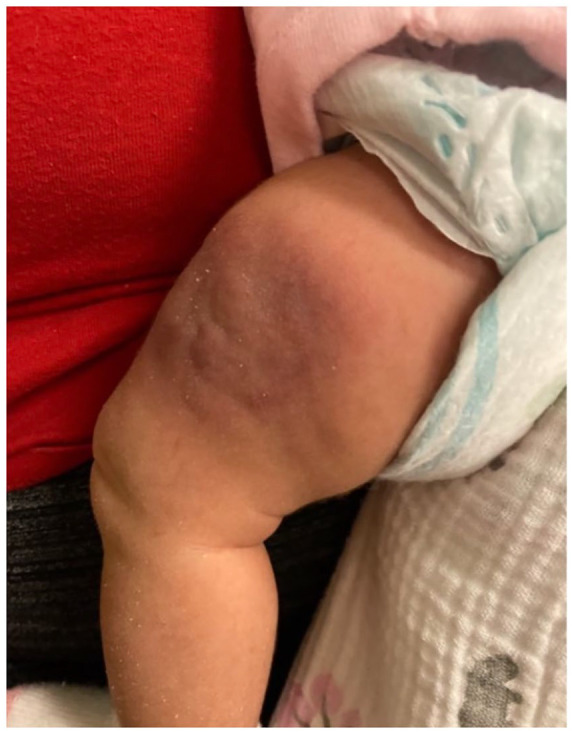
Circumscribed purpuric lesion on left anterolateral thigh.

Physical examination reveals a left thigh 4 cm dark purpuric papular lesion that was well circumscribed, firm, and slightly warm to touch (Figure 1). Bilateral femoral artery and dorsalis pedis pulses were intact, capillary refill was less than 2 seconds on each foot, and the affected leg had normal range of motion. The remaining physical exam was unremarkable. A complete blood cell count (CBC) demonstrated a white blood cell count of 11 000/μL (reference range, 7050-14 990/μL), hemoglobin of 10.8 g/dL (reference range, 8.9-12.7 g/dL), and platelet count of 220 × 10^3^/μL (reference range, 229-597 × 10^3^/uL). Ultrasound of the mass demonstrated thickening of the subcutaneous soft tissues and musculature of the anterolateral left thigh with irregular-shaped hypoechoic foci. Magnetic resonance imaging (MRI) of the left thigh showed a 2.4 × 0.9 × 3.9 cm conglomerate of T1 hypointensity with marked dermal thickening and hyperemia. Nine days after initial presentation, at a follow-up visit with her outpatient pediatrician, the patient’s labs showed a hemoglobin of 8.9 g/dL, platelet count of 273 ×10^3^ μL/mL, D-dimer of 4.40 μg/mL (< 0.40 μg/mL), and fibrinogen of 233 mg/dL (reference range, 220-240 μg/mL). Given the rapid growth of the mass with a decrease in hemoglobin, she was admitted to the hospital for further management.

## Discussion

### Hospital Course

The patient underwent daily monitoring of her CBC, fibrinogen, and D-dimer over the course of 4 days. The results showed a worsening anemia with a decrease in hemoglobin to 8.2 g/dL, thrombocytopenia of 101 × 10^3^ μL/mL, hypofibrinogenemia of 156 mg/dL, and increase in D-dimer to 7.88 μg/mL. These lab results were concerning for a life-threatening consumptive coagulopathy known as Kasabach-Merritt phenomenon (KMP). A biopsy of the lesion was obtained because imaging was not definitively diagnostic. The biopsy demonstrated infiltrating vascular nodules that were composed of spindle-shaped endothelial cells, as well as lymphatic vessels that appeared capillary hemangioma-like. Red cell extravasation was also seen, with scattered hemosiderin deposition and occasional microthrombi. On histopathologic staining, cells were negative for glucose transporter 1 (GLUT-1) and human herpesvirus-8 (HHV8), but diffusively positive for D2-40 within the spindles, consistent with a diagnosis of kaposiform hemangioendothelioma (KHE).

**Figure 2.**
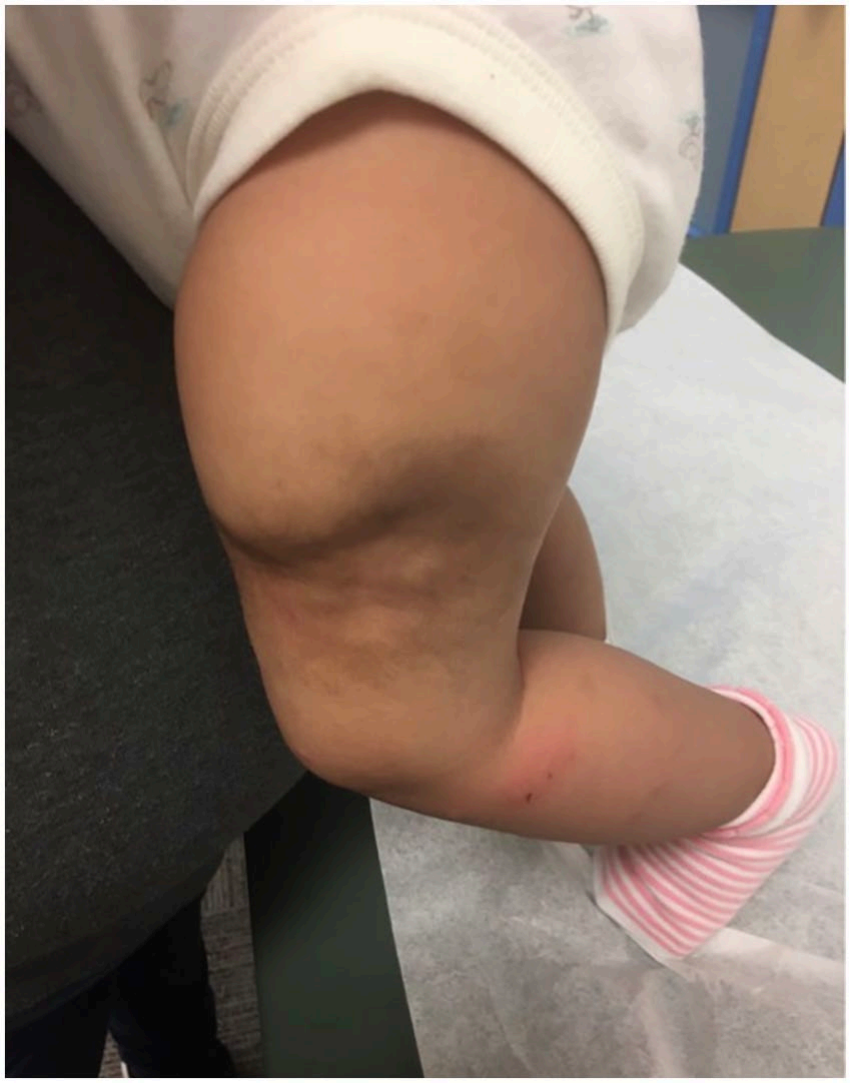
KHE after treatment.

After the biopsy confirmed the diagnosis of KHE in the setting of worsening KMP, the patient was treated in the inpatient setting for 4 days with oral prednisolone and sirolimus (an mTOR inhibitor), resulting in significant improvement in her consumptive coagulopathy. She was then discharged home and followed in the outpatient setting, where she continued to do well, and her consumptive coagulopathy eventually resolved after 3 weeks of medical treatment. Steroids were completely weaned at 2 months post discharge, with the plan to continue sirolimus for 2 years. Clinically, the patient showed continued improvement in pain, and the lesion became less firm. Scarring from the KHE led to significant deformation (Figure 2), for which the patient will need excision and reconstruction by plastic surgery at 4 to 5 years of age.

### Final Diagnosis

The combination of a rapidly enlarging mass, and the patient’s histological findings of D2-40 within spindle cells, confirmed the diagnosis of KHE. This patient’s course was also complicated by KMP, a triad of microangiopathic hemolytic anemia, thrombocytopenia, and consumptive coagulopathy, that is a potentially life-threatening condition associated with KHE and tufted angiomas.^1,2^

### Discussion of Case and Literature

Kaposiform hemangioendothelioma is a rare vascular tumor typically diagnosed in infancy or childhood. The cause and incidence are not well known. Due to the rarity of KHE, it is often under-reported or misdiagnosed; however, it is estimated to occur in 0.071 per 100 000 children.^1^ Retrospective reports have also indicated that KHE has a slight predilection for males.^3,4^

Presentation of KHE is variable, from a firm, palpable mass with cutaneous involvement, to mild or complete lack of cutaneous findings. Approximately, 12% of patients present with no cutaneous involvement.^1^ Diagnosis can be further complicated when noncutaneous lesions are located within the retroperitoneum, and are often misdiagnosed as other pediatric retroperitoneal tumors, such as neuroblastoma.^5^ Age of onset is usually in the first year of life, with a mean age of presentation at 2 months of age, and about half of cutaneous KHE lesions are noted at birth.^1^ The majority of cutaneous KHE lesions present as a rapidly enlarging, firm, purpuric mass and are often associated with KMP. Kasabach-Merritt phenomenon is characterized by a normal to slightly prolonged prothrombin time and partial thromboplastin time, severe thrombocytopenia, hypofibrinogenemia, and elevated D-dimer, and affects up to 71% of patients with KHE.^1,3^ When unrecognized or not treated promptly, KMP can cause severe bleeding, disseminated intravascular coagulation (DIC), shock, and multi organ failure. It is the most common cause of death in KHE.^3,4^ The presence and severity of symptoms is largely dependent on the location and size of the KHE. The majority of patients do experience pain, especially if KMP is present.^1^

Kaposiform hemangioendothelioma requires prompt diagnosis given its association with KMP, and involves clinical, laboratory, and radiographic evaluation to confirm the diagnosis. A platelet count, hemoglobin, D-dimer, and fibrinogen are critical in identifying KMP. Serial evaluation may reveal worsening anemia, thrombocytopenia, and a consumptive coagulopathy, including hypofibrinogenemia and elevated D-dimer.^3^ Ultrasound is often the first imaging modality utilized and can be helpful in characterizing the depth and size of the lesion, as well as distinguishing it from a cellulitis/abscess or other vascular or solid lesions. If the diagnosis of the lesion remains unclear despite ultrasonography, MRI of the affected area can be utilized to better characterize the lesion. If the diagnosis is clinically and radiographically unclear, as was the case for this patient, a biopsy is critical to confirm the diagnosis. If performed, tissue samples of KHE will demonstrate characteristic confluent nodules composed of spindle endo-thelial cells. Within the tissue, lymphatic channels contain cells, including red blood cells, platelet thrombi, and eosinophilic hyaline bodies.^3^ Staining is notable for positive vascular endothelial markers, such as CD31, as well as lymphatic endothelial marker D2-40, and negative for GLUT-1 and HHV8.

Management of KHE is largely supportive, and focuses on pain management, with many patients having mass effect secondary to tumor engorgement.^1,3^ Management recommendations are often based on expert opinion and case series, rather than large randomized control clinical studies.^1^ The Food and Drug Administration (FDA) has not approved the use of any medications specifically for KHE, and observation and supportive care are important in the absence of KMP. If KMP is present, supportive care may not be sufficient, and pharmacologic management is often needed due to the high risk of life-threatening bleeding with KMP. Medical therapies commonly include sirolimus, an mTOR inhibitor that downregulates the generation of abnormal blood vessels. Sirolimus may be used with or without systemic corticosteroids, which have an anti-inflammatory effect. In 2013, practice standards were created by a multidisciplinary effort in North America and consensus recommendation included a regimen of weekly vincristine and systemic corticosteroids.^6,7^ Recent recommendations now favor sirolimus plus steroids as the preferred first line of therapy for KHE complicated by KMP.^1^ When given in tandem, sirolimus and steroids showed faster improvement of KMP versus sirolimus monotherapy.^8^

For patients with KHE lesions that are life-threatening, small, or for those lesions that have already decreased in size with medical therapy, complete surgical resection offers the most definitive treatment. However, upon presentation, the majority of KHE tumors are inoperable due to infiltration into surrounding tissues, and due to the associated KMP. For patients with KHE complicated by KMP, the use of embolization is effective, but the potential side effects of skin necrosis and bleeding should be considered.^1,9^ A limitation of embolization therapy is that it can only be utilized when there is a single feeding vessel, and KHE is a highly vascularized tumor. Finally, platelet transfusions should be avoided unless the patient has active bleeding, and packed red blood cells are reserved for patients with symptomatic anemia, as transfusions can exacerbate intratumoral bleeding and lead to tumor growth and pain.

## Conclusion

This case emphasizes the critical importance of prompt recognition and treatment of KMP that is often seen with KHE. An infant presenting with a purpuric mass involves a wide differential, including cellulitis/abscess, atopic dermatitis, vaccine reaction, vascular malformation or tumor, or malignancy. An infectious etiology or vaccine reaction was less likely in this patient given lack of fever or palpable fluctuance on physical exam and the lack of leukocytosis on CBC. Continued bloodwork confirmed the presence of a consumptive coagulopathy which increased suspicion for an ongoing hematologic process, and decreased the likelihood of a vascular birthmark, congenital hemangioma, or venous malformation, diagnoses, which should all be considered in patients with enlarging purpuric lesions. Tufted angiomas are benign vascular tumors thought to lie on a spectrum with KHE and should also be considered. They can be differentiated from KHE on histopathological review by the size of their capillary lobules, which are smaller in comparison to KHE, and by their lack of spindle cells.^10^ While not always necessary for definitive diagnosis of KHE, biopsy of the lesion proved helpful in determining the final diagnosis and treatment plan for the patient.

Given the variety of causes for purpuric lesions in children and the rare nature of KHE, the diagnosis is easily and frequently missed, leading to potentially life-threatening delays in care in the presence of associated KMP. When examining children with purpuric lesions, clinicians should consider ultrasound as a first-line imaging modality to differentiate between infectious, hematologic, and oncologic etiologies. If KHE is suspected, serial bloodwork should promptly be pursued, and can lead to early identification of KMP. Long-term complications associated with KHE process include scarring, chronic pain, diminished range of motion or function, and lymphedema.^1^ Given the morbidity and possible mortality of KHE in the presence of KMP, clinicians should be familiar with its unique presentation, complications, management, and prognosis.

## Author Contributions

MS: Drafted the manuscript; Agrees to be accountable for all aspects of work ensuring integrity and accuracy. II: Critically revised the manuscript. PM: Critically revised the manuscript.
